# Impact of Parents' Oral Health Literacy on Their Own and Their Children's Oral Health in Chinese Population

**DOI:** 10.3389/fpubh.2022.809568

**Published:** 2022-03-08

**Authors:** Yu Wang, Marita R. Inglehart, Chao Yuan

**Affiliations:** ^1^Peking University School and Hospital of Stomatology, National Center of Stomatology, National Clinical Research Center for Oral Diseases, National Engineering Research Center of Oral Biomaterials and Digital Medical Devices, Beijing, China; ^2^International Trained Dentist Program, University of Michigan School of Dentistry, Ann Arbor, MI, United States; ^3^Department of Periodontics and Oral Medicine, School of Dentistry, University of Michigan, Ann Arbor, MI, United States; ^4^Department of Preventive Dentistry, Peking University School and Hospital of Stomatology, National Center of Stomatology, National Clinical Research Center for Oral Diseases, National Engineering Research Center of Oral Biomaterials and Digital Medical Devices, Beijing, China

**Keywords:** oral health literacy, parents, children, diet, behavior

## Abstract

**Background:**

Oral health literacy (OHL) has been recognized as a component of oral health disparities; however, the precise relationship between literacy and oral health outcomes has not been established. To explore the role of parents' OHL for their own subjective oral health, related behavior, and for the proxy assessment of their child's oral health, oral health-related behavior.

**Methods:**

Survey data were collected from 406 parents of 4- to 7-year-old children in Beijing, China. The background characteristics, oral health assessment, oral health-related behavior, knowledge and attitudes, and diet-related questions of parents and their children were surveyed by a questionnaire. OHL was assessed with the Hong Kong Rapid Estimate of Adult Literacy in Dentistry (HKREAL-30) Scale and a revised version that asked the respondents to indicate if they understood the words (HKREALD-30-Understand).

**Results:**

The HKREALD-30 responses correlated with the HKREALD-30-Understand responses. The higher the parents' HKREALD-30-Understand scores, the better they described the health of their own teeth and gums, the greater their child's diet was influenced by the protein, sugar and calories of the food, and the more positive their oral health-related attitudes were. The higher the parent's HKREALD-30 scores, the healthier they described their child's teeth and gums.

**Conclusions:**

Both the HKREALD-30 and HKREALD-30-Understand Scores correlate with parents' self and proxy oral health-related responses. Chinese parents could understand that the word would add predictive value to the prediction of how parents' oral health literacy affects their own oral health care, children's oral health and other related aspects.

## Introduction

Previous evidence has proven the prevalence of low literacy in health care settings and its adverse influence on health outcomes (1, 2). Low general health literacy is associated directly and indirectly with a range of poor health outcomes, not only because it is related to risk factors for poor health care but also because it affects patients' utilization of health care services, their cooperation with treatment recommendations, and their health-related behavior in general (3–6). Oral health-related literacy studies began later and were especially influenced by the fact that an oral health literacy instrument was developed in 2007, the Rapid Estimate of Adult literacy in Dentistry (REALD-30) (7).

One very important consideration was how parents' level of oral health literacy would affect their own oral health and the oral health of their children. Although the precise relationship between literacy and oral health outcomes has not been established (8–11), OHL has been increasingly recognized as a component of oral health disparities. Related studies revealed that the better parents' oral health literacy was, the better their own oral health (12, 13), and the better their children's oral health (10, 14). Low parental OHL was associated with low oral conditions among their children (9, 15–18). Studies have also demonstrated an association between decreased parental OHL and worse oral health behaviors, which had an adverse impact on children's oral health-related quality of life (19). Meanwhile, only a few studies have explored oral health-related behaviors. Common oral health-related behaviors such as brushing, flossing and regular dental visits need to be further evaluated.

Diet-related behavior has been studied extensively in the medical field. Because adults with limited literacy skills have difficulty interpreting and acting on health information that could reduce their risk factors and related symptoms, it is not surprising that people with low health literacy have poor disease management and diet-related behaviors in chronic illnesses (20, 21). Studies have also shown that diet-related attitudes are associated with health literacy and could predict diet-related behaviors and dietary quality (22). Less well educated, poorer and sicker people know less about where to look for dieted-related information and are less likely to attempt finding it (23). Therefore, we hypothesized that the poor oral health literacy population would not have a healthy diet-related attitude and behavior toward keeping their teeth healthy.

The importance of literacy in dentistry has attracted increasing attention. However, limited research has focused on oral health literacy among the Chinese population (24). The majority of oral health literacy instruments, such as REALD-30, were developed in English for North American contexts and cannot be used directly in Chinese-speaking groups, which limited the related studies. In 2012, Wong et al. (25) developed a measurement instrument, the Hong Kong Rapid Estimate of Adult Literacy in Dentistry (HKREALD-30), which is valid and reliable for the basic screening of oral health literacy among Chinese people. Therefore, it is a perfect time to utilize this instrument to see if oral health literacy in China would also be related.

Based on these considerations, the objectives of this study are to explore the role of parents' OHL in their own subjective oral health and related behavior and for the proxy assessment of their child's oral health, oral health-related behavior.

## Methods

This study followed the STROBE guideline checklist (26), with ethical approval obtained from the Peking University School and Hospital of Stomatology Ethics Committee (PKUSSIRB-201839139).

Data were collected from five hundred and one parent of children who participated in the regular dental examination, which was held in the daycare by dentists from Peking University School and Hospital of Stomatology in 2018. The parents were informed that a regular dental examination would be held at the daycare by dentists from Peking University School and Hospital of Stomatology and that an anonymous survey would be handed out after the examination. The parents who wanted to participate received the survey when they picked up their child, responded at home and returned the survey back the next day.

The survey mainly consisted of three variables: OHL, parent's oral health status, and children's oral health status. The detailed questionnaire is supplied in the Supplementary Materials. The variables extracted from the questionnaires were representative oral health assessment and related factors, which were further statistically analyzed with regard to their relationships with OHL score. Detailed information on the grading standard of each variable is presented in Supplementary Table 1. OHL was assessed by the HKREAL-30 Scale, which contains 30 Chinese words and was developed to assess oral health literacy among Chinese people. The parents were asked to check the words they could pronounce correctly and the words they understood the meaning. The mean score of each item and the mean sum score were calculated. The oral health status and related factors of parents and their children were assessed by their background characteristics, self-reported oral health, oral health-related behavior, knowledge and attitudes. The background characteristics included their gender, age, and years of education. Oral health-related behavior, knowledge and attitudes consisted of questions concerning parents' and their children's oral health, their frequency of brushing, flossing and dental visits, and the types of dental treatments they had received. The score ranges from 1 to 5, and a higher score indicates more positive. The mean and standard deviation of these questions were calculated. Furthermore, we survey the children's diet-related questions. The parents were asked to check on a list of 25 different foods and beverages that were often seen in the Chinese diet that their child had consumed during the past 24 hours. In addition, an open-ended question asked which other food their child had eaten in the past 24 hours; other questions inquired whether the child usually ate breakfast and how the costs and information about the food influenced their decision when ate out.

### Statistical Analysis

The parents' paper-pencil survey responses were entered into SPSS (Version 22). Descriptive statistics such as frequency distributions, percentages, means, standard deviations, and ranges were computed to provide an overview of the responses. Pearson correlation coefficients were used to determine the relationship between the constructs of interest. *P* < 0.05 was considered as significant.

## Results

There were 406 valid responses among 501 returned survey questionnaires. The respondents ranged in age from 26 to 48 years (Mean: 35.86, SD: 3.68). When the parents responded concerning the children's background characteristics, 52% were male, and the children ranged in age from 4 to 7 years (mean: 5.12, SD: 1.05). A detailed overview of the parents' and children's background characteristics is supplied in Supplementary Table 2.

Table 1 provides an overview of the respondents' answer scores concerning their own and their child's oral health and oral health-related knowledge, attitudes, and attitudes toward children's diet. The score ranges from 1 to 5, and a higher score indicates a more positive result. The parent self-reported health of teeth was 3.59, and their self-reported health of their gums was 3.67. Nearly 50% responded that they had very good or excellent oral health. Concerning that the proxy response was about their child's health of teeth and gums, the scores were 3.76 and 4.11, respectively. The absolute majority of the parents (83%) brushed their teeth more than once a day. However, only 15% flossed once per day or two times per day or more. Sixty-four percent of the children brushed their teeth two or more per day. However, only 5% flossed once per day to two times per day. The fluoride and sealant knowledge scores were 3.34 and 2.12, respectively.

**Table 1:** The mean score of parents' oral health-related responses, knowledge, attitudes, and attitudes toward children's diet.

		**Mean**	**SD**
Parents' oral health-related responses	Parent—self-reported health of teeth	3.59	0.93
	Parent—self-reported health of gums	3.67	0.88
	Parent—frequency of brushing teeth	4.83	0.39
	Parent—frequency of flossing teeth	1.82	1.20
	Child's health of teeth	3.76	1.00
	Child's health of gums	4.11	0.83
	Child—frequency of brushing teeth	4.54	0.72
	Child—frequency of flossing teeth	1.38	0.84
Parents' attitudes toward children's diet	Whether the food is healthy	3.80	1.12
	The calories	2.85	1.16
	The sugar content	3.02	1.23
Parents' oral health-related knowledge	Fluoride application	3.34	1.11
	Sealants application	2.12	0.99
Parents' oral health-related attitudes	Child's teeth clean	4.27	0.95
	Child's teeth healthy	4.17	1.08
	Child's teeth get brushed regularly	4.51	0.75
	Child visit dentist regularly	4.06	1.01
	Average oral health-related importance (Cronbach alpha = 0.834)	4.25	0.78

*The score ranges from 1 to 5, and a higher score indicates more positive*.

The parents' oral health-related attitudes were measured with four items that asked them how important it is for them that their child has clean teeth and healthy teeth, that the child's teeth are brushed regularly, and that the child sees a dentist regularly for check-up visits. Table 1 shows that the absolute majority of the parents held positive attitudes toward oral health.

Table 2 shows that 42% of parents had a dental visit in the past year, and on average, the majority had received checkups, cleaning, and fillings in the past. Concerning the children with dental treatment in the past, the data show that 87% had received a check-up in the past and 41% had fillings.

**Table 2:** Parents'/children's dental visit.

		**Parents (%)**	**Children (%)**
Dental treatment received in past:	Check-ups	65.8	86.9
	Cleanings	61.8	4.1
	Fillings	55.2	40.5
	Extractions	35.7	12.9
	Crowns	18.7	5.7
	Braces	9.4	2.6
	Root canal	24.1	7.5

The parents were asked how frequently their children had one of nine protein-containing foods on all five different carbohydrate-rich foods and seven sugar-rich foods. Table 3 shows that on average, the sum of protein-rich food on the day was 4.00 in the last 24 h, the sum of carbohydrate-rich food was 2.43, and the sum of sugar-rich food was 1.18. In addition, Table 1 indicates that the scores of whether the food was healthy, the calories, and the sugar content were 3.80, 2.85, and 3.02, respectively.

**Table 3:** Overview of the foods of children ate during the last 24 h.

		**Frequency of eating (%)**			**Mean**	**SD**	**Range**
		**0**	**1**	**2+>**			
Protein rich food	Beans	83.2	16.6	0.2	0.17	0.38	0–1
	Cheese	83.7	15.1	1.2	0.17	0.4	0–2
	Eggs	38.1	54.5	7.4	0.69	0.61	0–3
	Fish	77.5	21.2	1.3	0.23	0.44	0–2
	Formula milk	82.6	14.4	3.0	0.20	0.48	0–3
	Meat	31.0	45.2	23.8	0.98	0.84	0–3
	Milk	26.2	60.7	13.1	0.88	0.65	0–4
	Nuts	78.8	19.0	2.2	0.24	0.48	0–3
	Yogurt	43.1	51.1	5.8	0.64	0.61	0–3
	Sum of protein rich food				4.00	2.21	0–13
Carbohydrate rich food	Bread	70.8	25.2	4.0	0.33	0.54	0–2
	Cereal	94.4	4.6	1.0	0.06	0.27	0–3
	Crackers	59.1	37.1	3.8	0.46	0.59	0–3
	Noodles	56.6	41.2	2.2	0.45	0.54	0–2
	Rice	20.2	56.1	23.7	1.09	0.77	0–4
	Sum of carbo-hydrate rich foods				2.43	1.48	0–9
Sugar rich food	Cakes	56.8	38.0	5.2	0.48	0.6	0–3
	Candy	72.7	23.1	4.2	0.32	0.56	0–3
	Carbonated drinks	94.1	4.9	1.0	0.07	0.31	0–3
	Chocolate	83.0	14.9	2.1	0.18	0.44	0–3
	Dried fruits	83.1	15.6	1.3	0.18	0.43	0–3
	Energy drinks	96.7	3.1	0.2	0.03	0.17	0–1
	Ice cream	83.6	15.0	1.4	0.16	0.41	0–4
	Sum of sugar rich foods				1.18	1.25	0–8

To assess the parents' oral health literacy score, HKREAL-30 was provided twice. First, parents were asked if they could read each of the 30 words. Then, they were asked if they could understand each of the 30 words and understand the meaning. Table 4 shows that from these 30 items on average, the parents answered that they could read 23.91 and that they understood 17.08. Their responses concerning whether they could read the word and understand a word were significantly different in 17/30 words. Their reading and understanding overall scores correlated significantly and were equal to 0.44.

**Table 4:** Parents' responses concerning the Hong Kong Rapid Estimate of Adult Literacy in Dentistry (HKREAL-30) Scale.

**HKREAL-30**	**Can read the word**	**Understood the word**	***P-*value**
	**(*N =* 406)**	**(*N =* 406)**	
Smoking	0.88	0.83	0.636
Bacteria	0.88	0.82	0.552
Abscess	0.87	0.77	0.068
Bruxism	0.85	0.69	0.344
Diet	0.84	0.79	0.014
Implant	0.85	0.50	0.009
Dentition	0.78	0.40	<0.001
Hyperemia	0.84	0.70	0.194
Fracture	0.76	0.35	0.029
Genetics	0.81	0.75	0.052
Sedation	0.83	0.74	0.061
Diagnosis	0.83	0.75	0.033
Avulsion	0.79	0.49	0.002
Enamel	0.82	0.63	<0.001
Plaque	0.82	0.69	0.005
Malalignment	0.78	0.66	<0.001
Canine	0.85	0.59	<0.001
Occlusion	0.29	0.13	<0.001
Cyst	0.83	0.62	<0.001
Panoramic	0.73	0.17	0.405
Sealant	0.81	0.46	0.046
Socket	0.82	0.49	0.148
Cellulitis	0.74	0.19	0.382
Braces	0.74	0.23	0.785
Porcelain	0.84	0.69	<0.001
Orthodontics	0.81	0.68	0.003
Veneer	0.78	0.40	0.012
Erupt	0.78	0.47	<0.001
Fluoride	0.82	0.68	0.051
Molar	0.84	0.77	0.082
Sum score (Mean, SD)	23.91, 8.26	17.08, 8.80	<0.001

*P-value was calculated by t-test*.

The ultimate question of this manuscript is how oral health literacy is related to parents' and children's oral health, oral health-related behavior and parents' oral health knowledge and attitudes. Table 5 shows that when the number of words that the parents indicated they could read was significantly correlated with the child's subjective health of teeth and gums. The higher the reading oral health related literature score was, the healthier the parents described their child's health of teeth and health of gums, the more previous dental procedures that the parents had, but the fewer previous dental procedures the children had, the less sugar rich food the children ate, and the more important it was to the parents that they ate healthy food when they ate out, that they watched the calories when they ate out, and they watched the amount of sugar when they ate out. The better their reading-related oral health literacy score was, the less they thought that it is not true that fluoride can prevent caries, that it is not sure that sealant can prevent carriers, that they agreed that they know how to protect their child's teeth, and that they had positive attitudes.

**Table 5:** Relationships between parents' oral health literacy and their own and their child's subjective oral health and oral health-related behavior, knowledge and attitudes.

		**HKREAL-30 Read**	**HKREAL-30 Understand**
Parents' oral health-related responses	Parent—Subjective health of teeth^1^	0.01	0.10^*^
	Parent—Subjective health of gums^1^	0.00	0.11^*^
	Child—Subjective health of teeth^1^	0.19^***^	−0.10
	Child—Subjective health of gums^1^	0.16^**^	−0.10
Parents' responses concerning oral health-related behavior	Parent—Frequency of brushing teeth^2^	−0.07	−0.01
	Parent—Frequency of flossing teeth^2^	0.003	0.09
	Parent—Dental visit in past year	−0.08	−0.13^*^
	Parent—Sum of previous dental procedures^3^	0.13^*^	0.05
	Child—Frequency of brushing teeth^2^	−0.01	−0.02
	Child—Frequency of flossing teeth^2^	0.05	0.11^*^
	Child—Dental visit	0.01	0.03
	Child—Sum of previous dental procedures^3^	−0.16^**^	−0.03
Diet-related responses	Sum of protein rich food in past 24 h	−0.02	0.12^*^
	Sum of carbohydrate rich food in past 24 h	0.03	0.08
	Sum of sugar rich food in past 24 h	−0.11^*^	0.00
	Importance of healthy food when eating out^4^	0.16^**^	0.09
	Importance of calories when eating out^4^	0.11^*^	0.12^*^
	Importance of sugar when eating out^4^	0.18^***^	0.12^*^
	Child eats breakfast.	0.17^***^	0.03
Parents' oral health literacy, knowledge and attitudes	HKREALD-30- Understand	0.44^***^	1.00
	It is not sure that fluoride can prevent caries^5^	−0.12^*^	−0.01
	It is not sure that sealants can prevent caries^5^	−0.12^*^	−0.04
	Do you know how to protect your child's teeth?	0.13^*^	0.16^*^
	Oral health-related attitudes	0.19^***^	0.11^*^

*1. Answers Ranged From 1 = “Poor”, 2 = “Fair”, 3 = “Good”, 4 = “Very Good” to 5 = “Excellent”*.

*2. Answers Ranged From 1 = “Never”, 2 = “1 per Week or Less”, 3 = “3–4 per Week”, 4 = “1 per day”, 5 = “2 per day or More”*.

*3. The Parent—and the Child—Sum Score of Previous Treatments Was Computed by Adding 1 Point for Each of the Following Treatments the Parent or Child Had: Check-up, Cleaning, Filling, Extractions, Crowns, Braces, Root Canal*.

*4. Answers Ranged From 1 = not at all to 5 = Very Important*.

*5. Answers Ranged From 1 = “Disagree Strongly”, 2 = “Disagree”, 3 = “Neutral”, 4 = “Agree” to 5 = “Agree strongly”*.

* *P < 0.05*,

** *P < 0.01*,

*** *P < 0.001*.

When the parents' oral health literacy as measured with how many words they indicated they understood, the data showed that the more words they understood, the better the parents' objective health of their teeth and of gums, the more likely they had a dental visit in last year and the more likely their child's teeth get flossed, the more protein rich food their child had in the last 24 h, the more important it was to watch the calories and sugar when they ate out, and the greater they agreed that they knew how to protect their child's teeth and the more positive their oral health related attitudes.

## Discussion

The research contains 406 subjects, which is a good size that enabled us to test the hypothesis that we were interested in. The heterogeneity of the respondents' background characteristics was also satisfactory, as 125 fathers, which is a considerable number, participated in this research. There was a wide range in the educational degree. However, on the whole, the sample was highly educated, which may potentially limit the range of responses to oral health literacy skills, as research has proven that a higher education level is strongly associated with parents' oral health-related behavior and children's health of teeth (27–31). In addition, the income level range varies widely, meaning that the socioeconomic background of the parents ranges from a very low combined family income to a very high combined family income. Given that there is a strong relationship between oral health literacy and the family's socioeconomic status (32–36), the variety of socioeconomic backgrounds in our research would also provide us with a wide range of oral health literacy levels. There were approximately half males and half females, and they ranged in age from 4 to 7 years, with most children having primary dentition. The gender and age of our subjects also provide an ideal outlook for our study.

Parents' oral health behavior has been substantiated to affect their children's oral health behavior and oral health status (37). In our study, 83% of parents said they bush one to two times per day, meaning most of the parents were very good with brushing. However, the children did not brush as frequently as the parents. The frequency of flossing of both parents and children was very low. As reported by parents, we have a wide range of food-related responses, which allows us to look if all oral health literacy would be correlated with how much protein-, carbohydrate- and sugar-rich food they ate. The health-related attitude was evaluated by asking questions regarding when eating out what factors of parents considered important. However, only a low proportion of parents paid attention to the food composition. This phenomenon may be because of the features of Chinese food. The main components of Chinese meal are rice, flour, and various vegetables. Therefore, people are not used to pay attention to this aspect.

As shown in our study, parents' reading and understanding-based OHL was correlated with children's subjective health of teeth and gums and parents' oral health-related behaviors. The reading and understanding sum scores were correlated at the level of 0.44, which showed that what parents could read and what they understood were not necessarily correlated. When we have a closer look at each of the words we tested parents, we can see that the reading score and the understanding score of words: diet, implant, dentition, fracture, diagnosis, avulsion, enamel, plaque, mal-alignment, canine, occlusion, cyst, sealant, porcelain, orthodontics, veneer, and erupt are significantly correlated. There might be two circumstances that caused this result. The first was when the word was very uncommonly used in daily life, such as occlusion. People did not know either how to read or the meaning. Therefore, the two scores were significantly correlated. The second circumstance was that the word was composed of easy-reading characters, the term was used wildly, and even laypeople were familiar with them. Therefore, parents who could read the word also understood them. It is notable that Chinese is character-based rather than word-based, and a single character can be used as a word or meaning unit. This feature means that the examinee may read the word correctly without knowing its meaning, which is usually unlikely for English-speaking persons.

Having both reading and understanding skills measured could provide us with two different levels of inside. One would be how well they were trained to read symbols in Chinese. The other would be to understand the underlying meaning and what it means, which would be correlated with other aspects of their life. Therefore, they were both are significant. There are some findings that were completely different, such as the subjective health of the teeth and gums. The difference reveals that when parents truly have a deeper understanding, they might be better prepared to take care of their teeth and gums. Therefore, Chinese parents could understand that the word would add predictive value to the prediction of how parents' oral health literacy affects their own oral health care, children's oral health and other related aspects.

This study had some limitations. First, when testing respondents' reading-based oral health literacy, parents were asked to self-report. The accuracy of their pronunciation was not checked by one study, which might result in error. The second limitation was that the research was conducted in several university-affiliated childcare centers in the capital of Beijing. The overall educational level was high. With most parents working, the family income differed widely, but overall, it was definitely more weight toward people who potentially have higher literacy. As place of residency is also a significant factor that influences oral health-related quality of life (29), the sample was not a completely representative of the Chinese population. It would be worthwhile to look more into it in future studies, and a random sample across the spectrum of social demographic background or social status, such as low education and low-income populations, would be included.

Studies regarding health literacy in China are limited. From the handful of studies, we found that a low health literacy level in China may lead to the ignorance of risk factors and poor utilization of medical resources when having chronic diseases (38). Health literacy is the main factor affecting health promotion, and lower health literacy may lead to no treatment seeking and poor cooperation with the treatment recommendation (39, 40). It is warningly important for Chinese dentists to realize and understand the importance of oral health literacy to help patients with poor oral health literacy and ensure better oral health outcomes.

## Conclusions

Both HKREALD-30 and HKREALD-30-Understand Scores correlate with parents' self and proxy oral health-related responses. Chinese parents could understand that the word would add predictive value to the prediction of how parents' oral health literacy affects their own oral health care, children's oral health and other related aspects.

## Data Availability Statement

The raw data supporting the conclusions of this article will be made available by the authors, without undue reservation.

## Ethics Statement

The studies involving human participants were reviewed and approved by the Biomedical Ethics Committee of Peking University School and Hospital of Stomatology (PKUSSIRB-201839139). The patients/participants provided their written informed consent to participate in this study.

## Author Contributions

YW, MI, and CY conceptualized the study, performed the data analysis, wrote the manuscript, and critically revised the manuscript. CY collected the data. All authors participated in the design, interpretation of the studies, analysis of the data, and review of the manuscript.

## Funding

This study was funded by Peking University School and Hospital of Stomatology (Grant Number: PKUSSNCT-20Y02) and Peking University International Strategic Partnership Fund (Medical science, 2021).

## Conflict of Interest

The authors declare that the research was conducted in the absence of any commercial or financial relationships that could be construed as a potential conflict of interest.

## Publisher's Note

All claims expressed in this article are solely those of the authors and do not necessarily represent those of their affiliated organizations, or those of the publisher, the editors and the reviewers. Any product that may be evaluated in this article, or claim that may be made by its manufacturer, is not guaranteed or endorsed by the publisher.

## Abbreviations

OHL: Oral health literacy
HKREAL-30: Hong Kong Rapid Estimate of Adult literacy in Dentistry
HKREALD-30-Understand: Understand the words in the Hong Kong Rapid Estimate of Adult Literacy in Dentistry
REALD-30: Rapid Estimate of Adult literacy in Dentistry.
