# Supplementary material for: Impact of Parents' Oral Health Literacy on Their Own and Their Children's Oral Health in Chinese Population
Source: Front Public Health. 2022 Mar 8;10:809568. doi: 10.3389/fpubh.2022.809568 (PMC8957213; doi:10.3389/fpubh.2022.809568)
Supplement: Supplementary file 1 [file Data_Sheet_1.pdf]

Table of contents:

Supplementary Table 1
Supplementary Table 2
Questionnaire

Supplementary Table 1. Variables involved in the present study

	Categories	Subcategories	Options
**Oral health literacy**	**HKREAL-30**	-	Can read the word
	**HKREALD-30-Understand**	-	Understood the word
**Parents' oral health status and related factors**	**Parents' oral health status self-assessment**	Self-reported health of teeth	
		Self-reported health of gums	
	**Parents' oral health related behavior**	Frequency of brushing teeth	
		Frequency of flossing teeth	
		Dental visit	
	**Parents' oral health related knowledge**	Fluoride application	
		Sealants application	
	**Parents' oral health-related attitudes**	Child's teeth clean	
		Child's teeth healthy	
		Child's teeth get brushed regularly	The score ranges from 1 to 5, and a higher score indicates more positive
		Child visit dentist regularly	
	**Parents' attitudes toward children's diet**	Whether the food is healthy	
		The calories	
		The sugar content	
**Children's oral health status and related factors**	**Children's oral health status assessment**	Child's health of teeth	
		Child's health of gums	
	**Children' oral health related behavior**	Frequency of brushing teeth	
		Frequency of flossing teeth	
		Dental visit	

Supplementary Table 2: Overview of the parents' and children's background characteristics

	Background characteristics	Number	Percentages (%)
	Relationship to the child:		
	- Father	125	30.8
	- Mother	281	69.2
	Highest educational degree:		
	- Middle school	4	0.9
	- High	9	2.2
	- Technical school	2	0.5
	- Junior college	43	10.6
	- Bachelor	156	38.4
	- Master and/or above	192	47.3
Parents'	Employment:		
	- Working full-time	373	91.9
	- Working part-time	4	1.0
	- Stay-at-home	18	4.4
	- Other	11	2.7
	Combined family income/year:		
	- under 200k	137	35.4
	- 300k-500k	206	53.2
	- 500k-700k	26	6.7
	- 700k-8k	16	4.1
	- more than 900k	2	0.5
	Gender:		
Children's	- male	210	51.7
	- female	196	48.3

Questionnaire

Peking University School and Hospital of Stomatology—Parent Survey

Thank you very much for answering these questions!

Date: ______Year ____Month ____Day ID: □□□□

Child's Name: ________________

Child's Gender: ________________

Child's BD: _________Year _______Month _______Date

Child's Height: ________________

Child's Weight: ________________

Parent's age: ____________

1. What is your relationship to the child? （**Please choose only one answer**）

   1) □ Father 2) □ Mother
   3) □ Grandparent 4) □ Relative
   5) □ other

2. What is your employment status? （**Please choose only one answer**）

   1) □ Working full-time 2) □ Working part-time
   3) □ Seeking employment 4) □ Stay-at-home
   5) □ Other

3. What is your highest academic degree? （**Please choose only one answer**）
   1) □Elementary school 2) □ Middle school 3) □ High school 4) □ Technical school
   5) □Junior college 6) □ Bachelor 7) □ Master and/or above

<u>Here are some questions about parent's dental health and visits:</u>

4. How would describe the health of your teeth and gum? （Please choose only one answer for each question）

	1 Excellent	2 Very good	3 Good	4 Fair	5 Poor
1）Teeth ………	□	□	□	□	□
2）Gum ………	□	□	□	□	□

5. How often do you brush your teeth? （**Please choose only one answer**）

1) ☐ Never 2) ☐ 1x week or less 3) ☐ 3x-4x week
4) ☐ 1x day 5) ☐ 2x day or more

6. How often do you floss your teeth？ （**Please choose only one answer**）

1) ☐ Never 2) ☐ 1x week or less 3) ☐ 3x-4x week
4) ☐ 1x day 5) ☐ 2x day or more

7. Did you visit the dentist during the past year？ （**Please choose only one answer**）

1) ☐ Yes 2) ☐ No

8. If NO，what is the reason？ （**Please choose only one answer**）

1) ☐ I did not have time.
2) ☐ Dental treatments are too expensive.
3) ☐ I think all my teeth are good.
4) ☐ I have some small dental issues, but they do not bother me. I will have them taken care of when they become symptomatic.

9. Please think about the dental care you received in the past, and select all of them.

（**May choose more than one answer**）

1) ☐ Check-ups 2) ☐ Cleanings
3) ☐ Fillings 4) ☐ Teeth pulled
5) ☐ Crowns 6) ☐ RCT
7) ☐ Braces

<u>Here are some questions about the child's dental health and visits：</u>

10. How would describe the health of your child's teeth and gum？？ （**Please choose only one answer for each question**）

	1<br>Excellent	2<br>Very good	3<br>Good	4<br>Fair	5<br>Poor
1）Teeth ………	☐	☐	☐	☐	☐
2）Gum ………	☐	☐	☐	☐	☐

11. How often do your child's teeth get brushed ？ **(Please choose only one answer)**

1) ☐ Never 2) ☐ 1x week or less 3) ☐ 3x-4x week
4) ☐ 1x day 5) ☐ 2x day or more

12. How often do your child's teeth get flossed？ （**Please choose only one answer**）

1) ☐ Never 2) ☐ 1x week or less 3) ☐ 3x-4x week
4) ☐ 1x day 5) ☐ 2x day or more

13. Have you done any of the following? （**Please choose only one answer for each question**）

	5<br>Every day	4<br>Every week	3<br>Sometimes	2<br>Occasionally	1<br>Never
1）Help my child brush……	☐	☐	☐	☐	☐
2）Check after my child brushed	☐	☐	☐	☐	☐

14. How often did your child see a dentist in the past? （**Please choose only one answer**）

1) ☐ When there was a problem
2) ☐ 1x every 2 years
3) ☐ 1x every 1 year
4) ☐ 1x every 6 monts

15. Please think about what dental care your child has received in the past.（**May choose more than one answer**）

1) ☐ Check-ups 2) ☐ Cleanings
3) ☐ Fillings 4) ☐ Teeth pulled
5) ☐ Crowns 6) ☐ RCT
7) ☐ Dental care in the operating room 8) ☐ Braces

16. Why did you bring your child to a dentist? （**Please choose only one answer**）

1) ☐ Check-ups
2) ☐ Tooth pain
3) ☐ Saw something different

17. What dental care do you think your child would need?

1) ☐ Check-ups 2) ☐ Cleanings
3) ☐ Fillings 4) ☐ Teeth pulled
5) ☐ Crowns 6) ☐ RCT
7) ☐ Dental care in the operating room
8）☐ Fluoride and sealant
9) ☐ Braces

18. How often during the last 24 hours did your child have the following food?

Milk________times Yogurt_____times Cereal__________times
Carbonated drink___times Fruits__times Formula milk______times

Noodles_____times Cakes______times Soup__________times
Fish______times Bread_____times Crackers__________times
Beans______times Rice______times Dried fruits_____times
Eggs_______times French fries______times Ice cream_______times
Nuts______times Chocolate_______times Butter__________times
Candy_____times Cheese_____times Energy drinks____times
Meat such as pork, beef, and chicken__________times

Please list any other food you child ate in the past 24 hours：_________________

19. Does your child usually eat breakfast？（**Please choose only one answer**）

1) ☐ Yes 2) ☐ No

20. When you eat out，how much do the followings influence your decision：**(Please choose only one answer for each question)**

	1<br>Not important at all	2	3	4	5<br>Very important
1) Costs	☐	☐	☐	☐	☐
2) Whether the food is healthy	☐	☐	☐	☐	☐
3) Calories	☐	☐	☐	☐	☐
4) Sugar contains	☐	☐	☐	☐	☐

21. Please tell me for each of the following sentences how much you agree with it？**(Please choose only one answer for each question)**

	1<br>Disagree strongly	2	3	4	5<br>Agree strongl
1) My child has a toothache or pain currently	☐	☐	☐	☐	☐
2) My child's teeth hurt when she/he eats/drinks something cold or hot	☐	☐	☐	☐	☐
3) My child's teeth hurt when she/he eats/drinks something sweet.	☐	☐	☐	☐	☐
4) My child's teeth hurt when she/he bites/chews.	☐	☐	☐	☐	☐
5) My child has pain when she/he opens her/him mouth wide.	☐	☐	☐	☐	☐
6) My child sometimes wakes up at night with a toothache.	☐	☐	☐	☐	☐
7) My child sometimes has a toothache at school.	☐	☐	☐	☐	☐
8) My child sometimes misses a day of school because of a toothache.	☐	☐	☐	☐	☐
9) My child has a nice smile.	☐	☐	☐	☐	☐
10) My child is happy with her/his teeth.	☐	☐	☐	☐	☐
11) My child sometimes complains about her/his teeth.	☐	☐	☐	☐	☐

12) It is not sure that fluoride could prevent caries ☐ ☐☐☐ ☐

13) Sealant could prevent caries ☐ ☐☐☐ ☐

22. It is important to me **(Please choose only one answer for each question)**

	1<br>Disagree strongly	2	3	4	5<br>Agree strongly
1）that my child has clean teeth	☐	☐	☐	☐	☐
2）that my child has healthy teeth	☐	☐	☐	☐	☐
3）that my child's teeth get brushed regularly	☐	☐	☐	☐	☐
4）that my child sees a dentist regularly for check-up visits.	☐	☐	☐	☐	☐

23. Do you know what could protect your child's teeth？（**Please only choose one answer**）

1)☐ Yes 2)☐ No（**Please jump to question 25**）

24. Where did you acquire the knowledge？（**May choose more than one answer**）

1)☐ School 2)☐ Seminars

3)☐ Media（such as TV, newspaper） 4)☐ Social media

5)☐ Previous dental experiences 6)☐ Other

25. Please check the option that best represents the total combined income of your family during the last year（**Please choose only one answer**）

1) ☐ under 100k 2) ☐ 100k-200k 3) ☐ 300k-400k

4) ☐ 400k-500k 5) ☐ 500k-600k 6) ☐ 600k-700k

7) ☐ 700k-800k 8) ☐ 800k-900k 9) ☐ 900k-1M

10）☐ above 1M

Number:

Please read the following words, and check the one you can **read** or **understand. Do not guess.**

Score:	Please check the word you can read	Please check the word you understand
1. Smoking	☐	☐
2. Bacteria	☐	☐
3. Abscess	☐	☐
4. Bruxism	☐	☐
5. Diet	☐	☐
6. Implant	☐	☐
7. Dentition	☐	☐
8. Hyperemia	☐	☐
9. Fracture	☐	☐
10. Genetics	☐	☐
11. Sedation	☐	☐
12. Diagnosis	☐	☐
13. Avulsion	☐	☐
14. Enamel	☐	☐
15. Plaque	☐	☐
16. Mal-alignment	☐	☐
17. Canine	☐	☐
18. **Occlusion**	☐	☐
19. Cyst	☐	☐
20. Panoramic	☐	☐
21. Sealant	☐	☐
22. Socket	☐	☐
23. Cellulitis	☐	☐
24. Braces	☐	☐
25. Porcelain	☐	☐
26. Orthodontics	☐	☐
27. Veneer	☐	☐
28. Erupt	☐	☐
29. Fluoride	☐	☐
30. Molar	☐	☐
